# Cholera

**Published:** 1855-02

**Authors:** 


*Cholera.*—A history of the cholera of 1854, in Brooklyn, has just been published, (originally in the N. Y. Journal of Medicine,) in pamphlet form, by Joseph C. Hutchinson, M. D., Physician to the Brooklyn Cholera Hospital. It is a carefully written essay from an evidently close observer. Among the symptoms and and phenomena he mentions *contraction of the pupils* as occurring in a large number of cases, "and was regarded as a sufficient reason for an unfavorable prognosis." He denies that cholera is always preceded by premonitory diarrhœa. However it may have been elsewhere, cases have repeatedly occurred within our observation, where premonitory diarrhœa was entirely wanting. Dr. Hutchinson gives full minutes of six cases of this sort.

The depression of animal heat during collapse, and its return after death, are very well shown in the following table:

| Case. | Age. | Sex. | Time of observation before death | Temperature. Body. | Temperature. Atmosphere. | Time of death. | Time of observation after death. | Temperature. Body. | Temperature. Atmosphere. |
|---|---|---|---|---|---|---|---|---|---|
| 1 | 33 | male | 9 h. A. M. | V'y decided coldness of surface. | 79° | 2 h. 30 m. P. M. | 8 h. P. M. | Axilla, 90° Perineum, 93° Over the heart, 90° Toes, 83° Fingers, 85° | *74° |
| 2 | 18 | Female | July 6th, 10 h. 30 m.P.M. | Axilla, 92° Calf of leg, 87° Side of abdomen, 49° Vulva, 49° Mouth, 78° | 80° | July 7th, 1 h. P. M. | 5 h. 30 m. P. M. | Side of chest 94° | 85° |
| 3 | 34 | Female | July 9th, 11 h. P. M. | Coldness very marked | 73° | July 10th, 8 h. P. M. | 8 h. 30 m. P. M. | Mouth, 81° Axilla, 81° Vagina, 91° Lower extremities 72° | 75° |
| 4 | 45 | Female | July 27th, 4 h. P. M. | Hand, 80° Foot, 83° Side of chest 100° | 84° | July 28th, 6 h. 30 m.A.M. | 8 h. 30 m. P. M. | Hand, 83° Foot, 78½° Side of abdomen, 88° | †74° |
| 5 | 36 | Female | 10 h. A. M. | Surface quite cold. | 80° | 7 h. P. M. | 10 h. P. M. | Pelvic cavity, 104° | 74° |
| 6 | 30 | Female | 11 h. 30 m. A. M. | Coldness very decided | 86° | 3 h. P. M. | 4 h. P. M. 9 h. P. M. | Axilla, 104° Pelvic cavity, 107° | 87° 87° |

* *Rigor mortis* well marked. † *Rigor mortis* well marked.

Dr. Hutchinson mentions an unnatural amount of blood in the membranes of the brain, with "more or less sub-arachnoid effusion of clear or reddish fluid." The ventricles had an unusual quantity "sometimes clear and at other times bloody."

We cut out, also, Dr. Hutchinson's conclusions, leaving our readers to digest them:

"1st. If we may draw a general inference from the observations made in the hospital in this city, cholera should be regarded as a much less fatal disease than it is usually conceded to be.

"2d. That premonitory diarrhœa, though usually, *does not invariably* precede the development of cholera.

"3d. The copious vomiting and purging of a fluid resembling rice-water, instead of constituting the essence of the disease, should, if not involuntary, be regarded as an indication of its favorable termination.

"4th. In the treatment of cholera, we should exhibit the highest respect for the *vis medicatrix naturæ.* I would rather be deprived of all other remedies, for this disease, than ice and beef-tea." H.

